# Characteristics of *Shisa* Family Genes in Zebrafish

**DOI:** 10.3390/ijms241814062

**Published:** 2023-09-14

**Authors:** Yansong Liu, Na Du, Beibei Qian, Congcong Zou, Zhouxin Yu, Fei Xu, Lijuan Wang, Sishi Qin, Feng You, Xungang Tan

**Affiliations:** 1 School of Marine Science and Engineering, Qingdao Agricultural University, Qingdao 266109, China; liuyansong5@163.com (Y.L.);; 2 CAS and Shandong Province Key Laboratory of Experimental Marine Biology, Center for Ocean Mega-Science, Institute of Oceanology, Chinese Academy of Sciences, Qingdao 266071, Chinayoufeng@qdio.ac.cn (F.Y.); 3 Laboratory for Marine Biology and Biotechnology, Pilot National Laboratory for Marine Science and Technology (Qingdao), Qingdao 266237, China; 4 University of Chinese Academy of Sciences, Beijing 10049, China

**Keywords:** gene expression, knockdown, CRISPR/Cas13d, *shisa-2*, *mesp-ab*, somite formation

## Abstract

*Shisa* represents a type of single-transmembrane adaptor protein containing an N-terminal cysteine-rich domain and a proline-rich C-terminal region. Nine *shisa* subfamily genes have been proposed in most vertebrates; however, some might be species-specific. The number of *shisa* genes present in zebrafish remains unclear. This study aimed to investigate the evolutionary relationships among *shisa* family genes in zebrafish (TU strain) using phylogenetic and syntenic analyses. The function of *shisa-2* was preliminarily examined via CRISPR/Cas13d-mediated knockdown. Following identification in zebrafish, 10 *shisa* family genes, namely *shisa-1*, *2*, *3*, *4*, *5*, *6*, *7*, *8*, *9a*, and *9b*, were classified into three main clades and six subclades. Their encoding proteins contained a cysteine-rich N-terminal domain and a proline-rich C-terminal region containing different motifs. A specific syntenic block containing *atp8a2* and *shisa-2* was observed to be conserved across all species. Furthermore, all these genes were expressed during embryogenesis. *Shisa-2* was expressed in the presomitic mesoderm, somites, and so on. *Shisa-2* was identified as a regulator of the expression of the somite formation marker *mesp-ab*. Overall, our study provides new insights into the evolution of *shisa* family genes and the control of *shisa-2* over the convergent extension cells of somitic precursors in zebrafish.

## 1. Introduction

To maintain their development and growth, cells must coordinate and integrate multiple functional modules, including controlling cytoskeleton remodeling, adapting to environmental alterations, and gene expression [1]. Functional modules are regulated by a series of signaling networks that communicate with each other via the interaction of their components with adaptors, docking, anchoring, or scaffold proteins [1,2,3]. Scaffold proteins contain several modular domains, including the SH3, WW, SH2, and PDZ domains, which mediate protein-protein interactions and/or protein-membrane associations [1,3].

The *shisa* family proteins are recently discovered adaptor proteins [4]. The first *shisa* family gene was discovered in 2005 and was named *shisa* because of its effect on the formation of African clawed frog (*Xenopus laevis*) heads [5]. As adaptor proteins, *shisa* family proteins play various roles in different species and developmental processes, including cancer development and apoptosis [4]. Shisa-1 inhibits Wnt receptor Frizzled glycosylation and fibroblast growth factor receptor (FGFR) phosphorylation during *X. laevis* head formation [5]. During somitogenesis, *X. laevis* Shisa-2 regulates segmental patterning by inhibiting Wnt receptor Frizzled glycosylation and FGFR phosphorylation [6]. During the growth and development of mouse commissural axons, SHISA-2 only inhibits the glycosylation of Frizzled 3 [7]. In C2C12 cells, SHISA-2 regulates the rearrangement of the muscle cytoskeletal actin F-actin and subsequently promotes myoblast fusion [8]. SHISA-3 serves as a tumor suppressor by accelerating catenin degradation [9]. SHISA-4–6 and shisa-8 can degrade proteins by regulating the ubiquitination of target proteins [4]. SHISA-5 can degrade the nonstructural protein 5 (NS5A) of the hepatitis C virus (HCV) [10]. Its binding to Cyclin B results in DNA damage and degradation [11]. SHISA-7 can transport γ-aminobutyric acid type A receptors (GABA_A_ receptors) during brain neurodevelopment in mice [12]. SHISA-6–9 are critical subunits of the α-amino-3-hydroxy-5-methyl-4-isoxazole propionic acid receptor during its biogenesis and function in the central nervous system [13,14]. Therefore, different *shisa* proteins play different roles in various biological processes, even though the same protein plays different roles in various tissues or cells with different characteristics [15] and may have tissue- and cell-specific regulatory pathways.

All proteins in this family contain a predicted signal peptide, an N-terminal cysteine-rich domain (Cys), a transmembrane domain (TM), and a C-terminal proline-rich region. In the C-terminal proline region, other motifs and/or domains, such as the PY ([LP] P×Y) motif, the PDZ (postsynaptic density protein) domain binding motif ([ST]×[VLI]), and the GRID domain (GABA_A_ receptor binding domain), are present [4,12]. Different *shisa* proteins play different roles in various species and developmental processes based on their domains and/or motifs [4]. In mice, SHISA-7 binds with GABA_A_ receptors through the GRID domain to control benzodiazepine actions [12]. The proline region of SHISA-5 (Scotin) maintains its endoplasmic reticulum (ER) specific expression [16]. SHISA-5 induces apoptosis through the interaction of its cysteine domain with Cyclin B [11]. The interaction of the TM and proline region of SHISA-5 with HCV NS5A is required to control NS5A degradation [10].

To date, nine *shisa* subfamily genes have been identified [4]. Most vertebrate genomes contain at least eight *shisa* genes (*shisa-2*–*9*). However, *shisa*-*1* is found only in zebrafish (*Danio rerio*) and *X. laevis* [17]. All of the subfamilies were predicted in zebrafish using the genome database and RNA sequencing; however, *shisa*-*8* was not found [4]. The zebrafish, a small freshwater teleost, is a widely used animal model in developmental biology, ecotoxicology, cancer, and neuroscience research. As a model organism, zebrafish have several advantages, such as a short breeding cycle, high fecundity, in vitro and transparent embryonic development, and high genetic homology to humans. However, the number of *shisa* family genes in zebrafish is still unknown. This study aimed to examine the presence of *shisa* family genes and their expression patterns in zebrafish and determine the potential functions of one of them during embryonic development. The findings could provide a basis for understanding the evolution of the *shisa* family genes and the function of related genes.

## 2. Results

### 2.1. Nine Shisa Subfamilies Identified in Zebrafish

**Figure 1:**
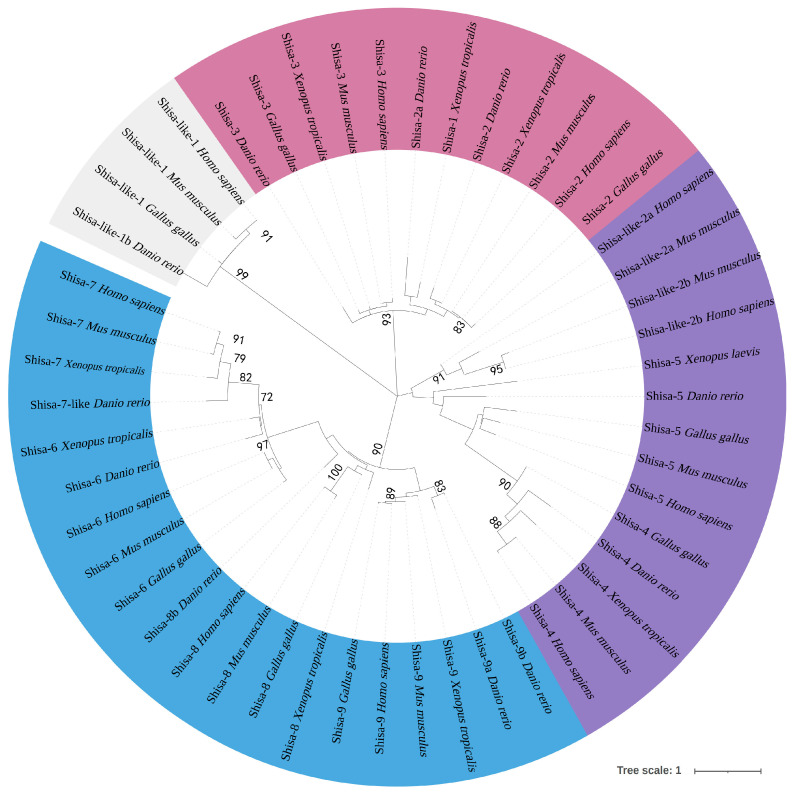
Phylogenetic tree of SHISA proteins. The phylogenetic tree was constructed via MEGA7.0 [18] using the Maximum likelihood method with 1000 bootstrap replicates. The GeneBank accession numbers for these genes are listed in Table 1. Notes: Confidence values higher than 70 are indicated in the tree.

In the zebrafish genomic and transcriptomic databases, 11 predicted *shisa* candidates were found and named *shisa 1bl*, *2*, *2a*, *3*, *4*, *5*, *6*, *7l*, *8b*, *9a*, and *9b* (Table 1). All of them were cloned and sequenced to verify the original sequences and names. After sequencing, all the encoded amino acids were used for phylogenetic analysis (Figure 1). As a result, 10 of them were classified into nine subfamilies that were consistent with the previous subfamily classifications, including Shisa-1, Shisa-2, Shisa-3, Shisa-4, Shisa-5, Shisa-6, Shisa-7, Shisa-8, and Shisa-9. The predicted Shisa-2a, Shisa-7l, and Shisa-8b were determined to be Shisa-1, Shisa-7, and Shisha-8, respectively (Table 1). The nine subfamilies were divided into three clades and six subclades (Figure 1). Shisa-1 and Shisa-2, Shisa-6 and Shisa-7, and Shisa-8 and Shisa-9 belonged to separate subclades.

### 2.2. Conserved Domain among shisa Proteins

**Figure 2:**
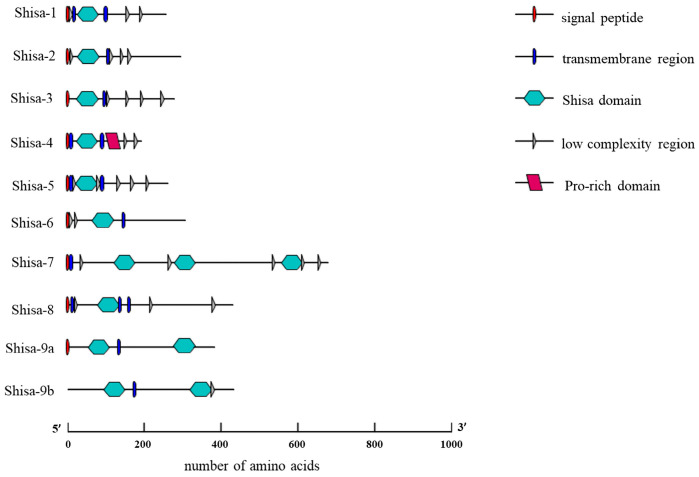
Domain architecture of zebrafish Shisa proteins.

**Figure 3:**
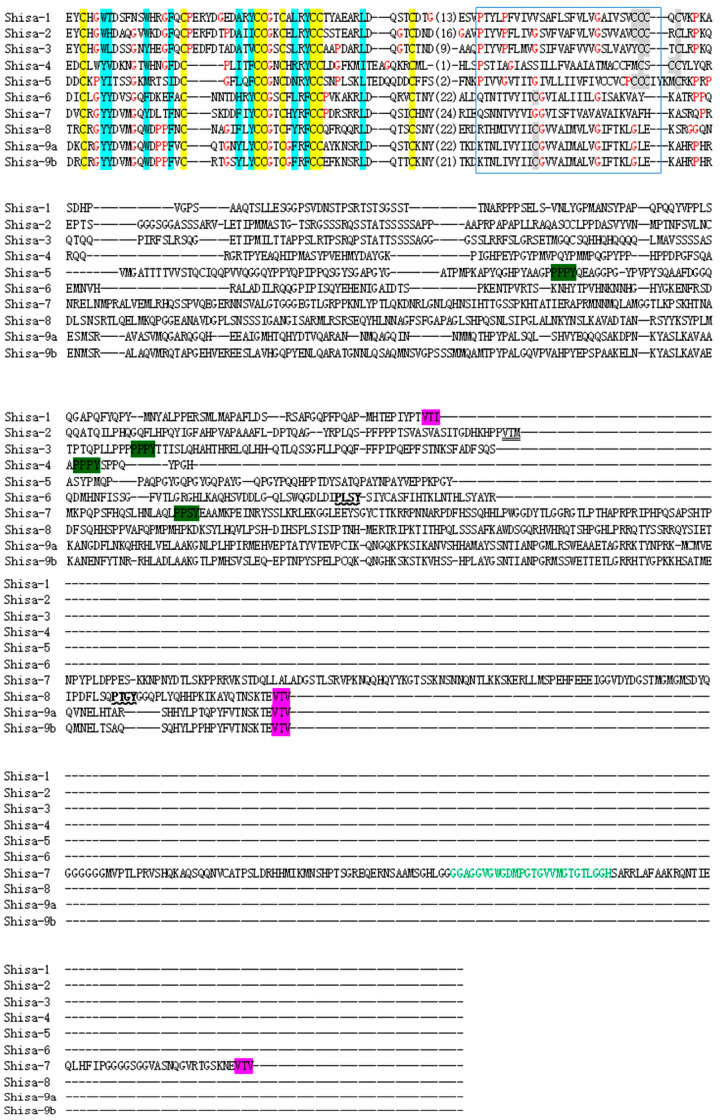
Sequence alignment of zebrafish Shisa proteins. A typical conserved domain (“C * C * CC * C * CC * C”, shaded yellow) of *shisa* protein and TM domain was observed. The number in sequence represents the amino acid number between two amino acids. The predicted transmembrane region is framed with a blue border (predicted by DNAMAN). Prolines and glycines are shown in red letters. The noncharged residues at the main hydrophobic residue positions are colored blue. The predicted cysteine residues within and after the transmembrane segment are marked in gray. PY motif ([LP]P×Y) are marked in green [4]. C-terminal PDZ-binding motifs([TSVYF]×[VIL]) are marked in pink [4]. PY-like motif, ‘
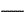
’; C-terminal PDZ-binding-like motif, ‘
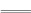
’. GRID domain [12] is shown in green letters.

The Shisa motif, TM, and low-complex proline-rich regions were predicted in all *shisa* proteins (Figure 2). Signal peptides were present in zebrafish Shisa proteins except for Shisa-9b. The proline-rich domain was only found in Shisa-4. The amino acid sequences were further aligned and analyzed (Figure 3). *Shisa* family proteins shared a low average amino acid identity of 17.48%. All of them contained a cysteine-rich domain in the N-terminus with the following distinct pattern: C * C * CC * C * CC * C (“*” represents a series of amino acid residues). Several cysteine residues were present near the C-termini of the predicted TMs in zebrafish Shisa-1–5, which were not found in Shisa-6–9 (Figure 3). Compared with other Shisa subfamilies, several sequence features present in Shisa-1–3; for example, long insertions with a “PE××D××DA” signature were observed between the second and third conserved cysteines in the cysteines-rich domain and a conserved sequence “P×××P” was found at the beginning of their predicted TMs (Figure 3) [4].

### 2.3. Shisa Genes Localization on Chromosomes

**Figure 4:**
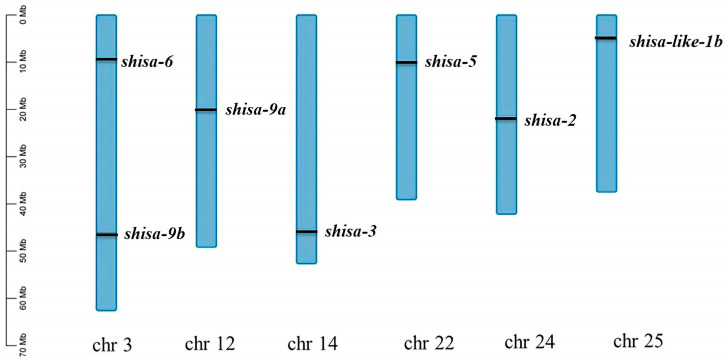
Chromosomal location of zebrafish *shisa* family genes.

The chromosomal locations of the Zebrafish *shisa* family genes are shown in Figure 4. Most genes were located on chromosomes 3, 12, 14, 22, 24, and 25; however, *shisa-1* (previously predicted to be *shisa-2a*), *shisa-4*, *shisa-7*, and *shisa-8b* were not located on those chromosomes based on the present genomic data.

### 2.4. Conserved Syntenic Block Containing Shisa-2

**Figure 5:**
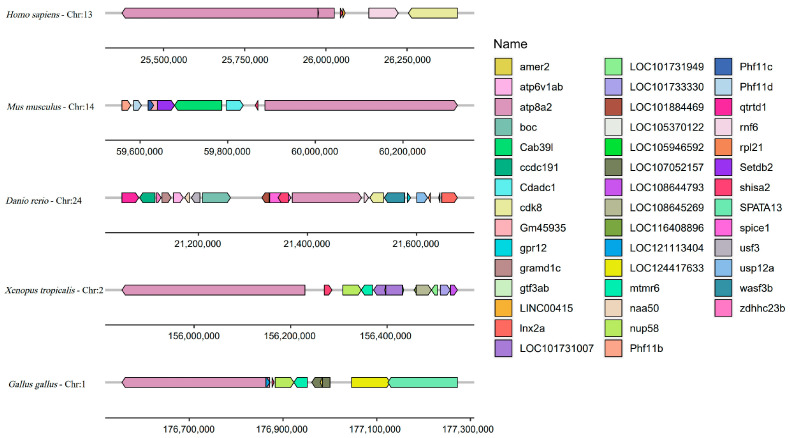
Synteny analysis of *shisa-2* neighboring genes in different species that share the same ancestral species.

Collinearity analysis was performed by comparing *shisa-2* chromosome distribution among *Homo sapiens*, *Mus musculus*, *Gallus gallus*, *X. tropicalis*, and *Danio rerio*. We found that a specific syntenic block containing *atp8a2* (ATPase phospholipid transporting 8A2) and *shisa-2*, arranged in reverse order, was conserved across all species (Figure 5).

### 2.5. Different Expression Patterns of Shisa Family Genes during Embryonic Development

**Figure 6:**
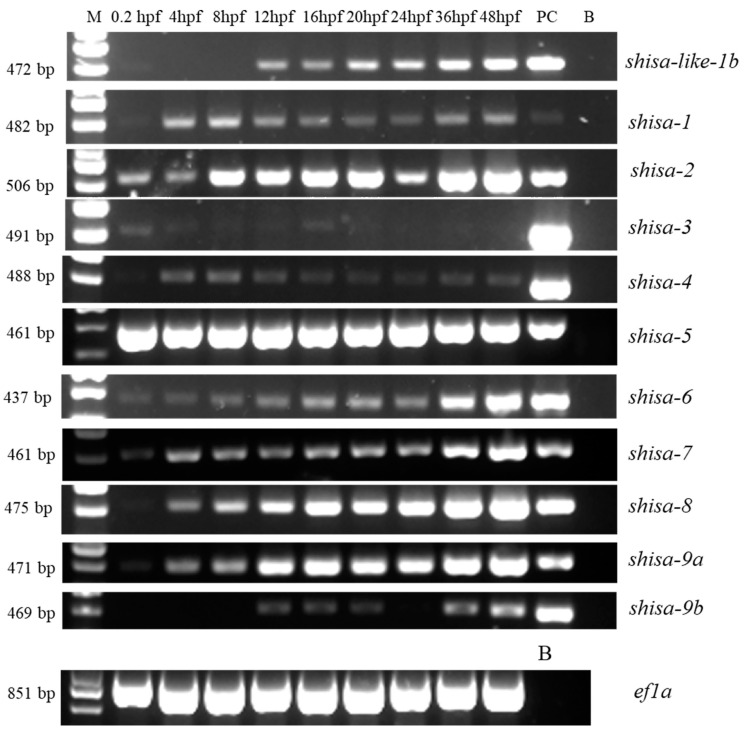
Temporal expression patterns of zebrafish *shisa* genes during embryonic development. B, blank. PC, positive control. M, marker.

The temporal expression profiles of *shisa* family genes in zebrafish during embryonic development were determined using semi-quantitative PCR (Figure 6). The results indicated that most *shisa* genes were expressed during zebrafish embryogenesis, and their expression was upregulated during embryonic development. Most of them were expressed at 0.2 h post fertilization (hpf), except *shisa-9b*. The expression of *shisa-3* was weak at 8 hpf, then increased. After 16 hpf, the expression decreased. *Shisa-9b* was expressed from 12 hpf. The transcripts of *shisa-1*, *shisa-2*, *shisa-4*, *shisa-5*, *shisa-6*, *shisa-7*, *shisa-8*, and *shisa-9a* were high or weak in all test stages.

**Figure 7:**
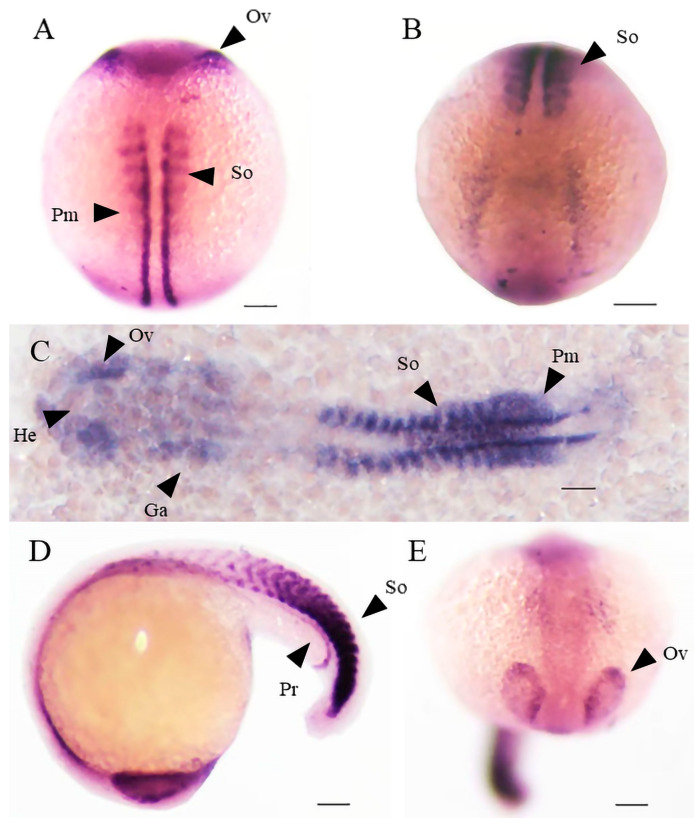
Expression pattern of zebrafish *shisa-2* during early embryonic development. *Shisa-2* transcript was detected in He (Head region), So (Somite), Ov (Optic vesicles), Pm (Pre-somatic mesoderm), Pr (pronephros), and Ga (Gill arch). (**A**,**B**), 11 hpf. (**C**), 16 hpf. (**D**,**E**), 20 hpf. (**A**), Head to Top. Dorsal View. (**B**,**E**). Dorsum to Top. Front View. (**C**), Head to left. Dorsal View. (**D**), Head to left, side view. Bar, 50 μm.

The spatiotemporal expression map of *shisa-2* was analyzed using in situ hybridization in zebrafish. At 11 hpf, *shisa-2* was expressed in the presomitic mesoderm, both in the medial and lateral regions of the somites and at the base of the eye vesicle (Figure 7A,B). At 16 hpf, *shisa-2* expression was strong in the somites, optic vesicles, gill arches, and head regions (Figure 7C). At 20 hpf, *shisa-2* expression in the differentiated somites gradually decreased but remained strong in the newly formed somites and presomitic cells in the tail region, pronephros, and optic vesicles (Figure 7D,E).

### 2.6. Abnormal Expression of mesp-ab by shisa-2 Knockdown

**Figure 8:**
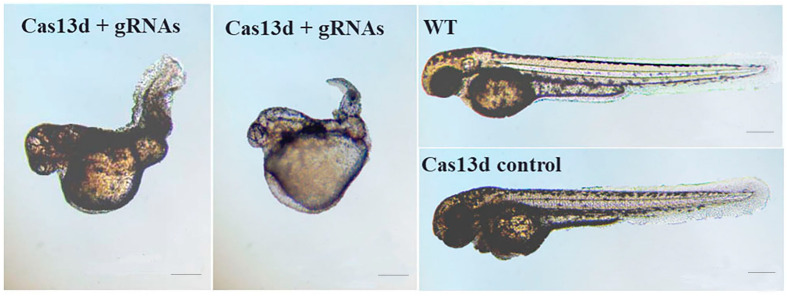
Phenotype of zebrafish embryos (48 hpf) after *shisa-2* knockdown. Bar: 200 μm, Magnification: 4×.

The phenotype of embryos and larvae injected with Cas13d mRNA and gRNA was assessed at 48 hpf (Table 2). Approximately 70% of the embryos co-injected with Cas13d mRNA and gRNAs at least three times had morphological malformations with curved and shrunken trunks (Figure 8). No morphological change was found in the embryos injected with Cas13d mRNA or gRNAs alone. As a result, the observed morphological changes were likely a specific phenotype of *shisa-2* knockdown (Figure 8). To confirm the specific phenotype, a rescue experiment using flounder *shisa-2* mRNA was performed. In that experiment, the percentage of embryos with severely curved trunks decreased, and also some rescued embryos displayed a weakly curved trunk (Supplementary Figures S1 and S2).

**Figure 9:**
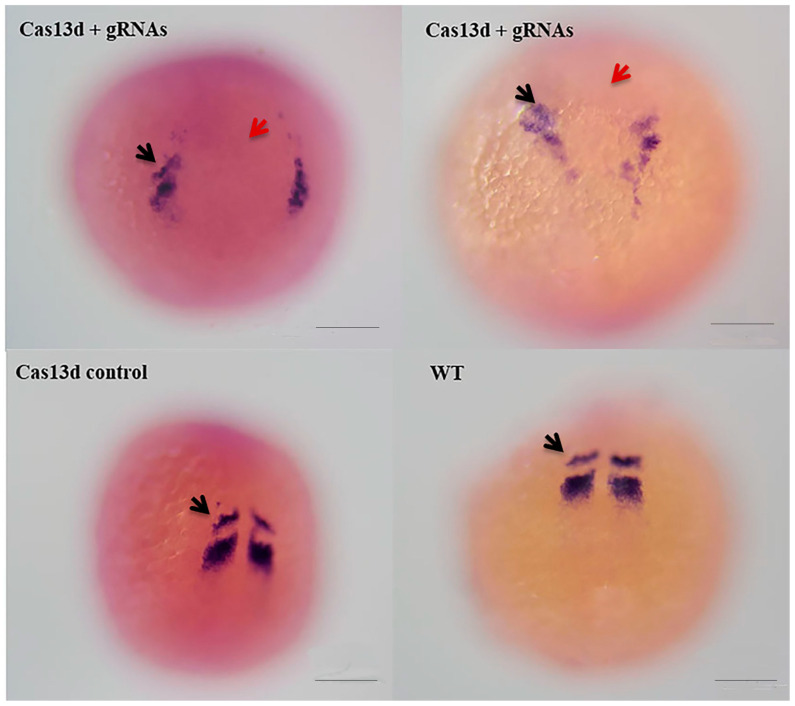
Expression of *mesp-ab* in embryos injected with Cas13d mRNA +gRNA, Cas13d mRNA, and no-injected embryos (wild type). Black arrows represent the *mesp-ab* signal. Red arrows indicate the space between the left and right stripes of *mesp-ab*. Head to top, dorsal view. Bar, 100 μm.

Because the embryonic trunk was abnormal after knockdown, somite formation was analyzed using the *mesp-ab* expression-somite formation marker gene. The results of in situ hybridization at 11 hpf demonstrated that the signal of *mesp-ab* expression was scattered and not clustered as a stripe (Figure 9) in the newly formed somites in the knockdown group. In contrast, two stripes were observed in the control groups. In addition, the space between the two *mesp-ab* positive group cells was wider than that of the control groups (Figure 9), indicating that the convergent extension of somitic precursors was delayed.

## 3. Discussion

As adaptor proteins, *shisa* family proteins play important roles during animal development. However, the number of *shisa* family genes present in zebrafish remains unclear. Additionally, their expression patterns and roles during zebrafish embryonic development are still unknown. In this study, 10 *shisa* family genes were identified in zebrafish and classified into the following nine subfamilies: *shisa-1* (previously named *shisa 2a*), *shisa-2* (*shisa 2*), *shisa-3* (*shisa 3*), *shisa-4* (*shisa 4*), *shisa-5* (*shisa 5*), *shisa-6* (*shisa 6*), *shisa-7* (previously named *shisa-7-like*), *shisa-8* (predicted name *shisa-8b*), and *shisa-9* (*shisa 9a and shisa 9b*). A specific syntenic block containing *atp8a2* and *shisa-2* was found to be conserved across all species. We also found that *shisa-2* was a regulator of the convergent extension cell movement of the somitic precursors during embryonic development.

### 3.1. Different Evolutionary Processes for Shisa Subfamily Genes

In this study, *shisa-1* was first confirmed in zebrafish. Vertebrates have at least nine *shisa* genes. *shisa-2–9* were discovered in all tested species. *Shisa-1* was not presented in amniotes and has been reported only in *X. laevis*, zebrafish, and *Oncorhynchus mykiss* [4,17]. *Shisa-1* and *shisa-2* might have been produced from genome duplication as they were in the same subclade. African clawed frogs and fish are known to have gone through tetraploidization or whole genome duplication [17]. Other studies [4,17] and our results indicated that the *shisa-1* subfamily was separated from subfamily *shisa-2*, which implied that *shisa-1* did not originate from tetraploidization or whole genome duplication. However, putative *shisa-1* genes in birds and the elephant shark *Callorhinchus milii* had been predicted in their genomic sequences [4]. It is more likely that the *shisa-1* gene degenerated in mammals. As a result, the occurrence of *shisa-1* and *shisa-2* genes might be due to the gene duplication–complementation–degeneration model [17]. Additionally, we found a specific syntenic block containing *atp8a2* and *shisa-2* conserved across all species. The *atp8a2* is considered an evolutionarily conserved gene [19]; therefore, *shisa-2* might be the original subclade gene of the *shisa-1* and *shisa-2* subfamilies.

*Shisa-8* is considered a mammalian-specific gene duplication of *shisa-9* [4]; however, we have isolated it in zebrafish for the first time. Our findings further suggest that *shisa-8* and *shisa-9* might have originated from genome duplication. In zebrafish, subfamily-specific gene duplications have been observed in two *shisa-9s* (*shisa-9a* and *shisa-9b*). In fish, many genes are found with duplicated gene phenomena, including pax3a/3b and pax7a/7b in zebrafish, medaka (*Oryzias latipes*), tetraodon (*Tetraodon nigroviridis*), fugu (*Takifugu rubripes*), and olive flounder (*Paralichthys olivaceus*) [20,21,22,23]. An additional specific genome duplication (FSGD) occurred in the teleost lineage before the beginning of teleost radiation [24]. Two different *shisa-9* genes are more likely to originate from fish-specific genome duplication.

### 3.2. Conserved Domains and Divergent C-Terminal Regions of Shisa Proteins

Apart from the conserved cysteine-rich domain of *shisa* family proteins in the N-terminus, several cysteine residues were present near the C-termini of the predicted TMs (Figure 3) of zebrafish Shisa-1–5, which have been proposed as potential sites for lipid modifications, such as palmitoylation [25]. Such modifications stabilize the protein and transport it to specific membranes [25]. Therefore, zebrafish Shisa-1–5 might be involved in cellular signaling or membrane trafficking through the modification. Subfamily Shisa-6–9 might not have a specific function, for there were no cysteine residues for lipid modification near the C-termini of their predicted TMs. Through further analysis, we found that these cysteine residues are regarded as the subfamily signatures, with “CCC[KQ]C”, “CCCRC”, and “YCCTC” motifs [4] in zebrafish Shisa-1, Shisa-2, and Shisa-3, respectively (Figure 3), although these cysteine residues were arranged as “CC[FR]*CSCC” and “C**C[SP]CC*****C” in Shisa-4 and Shisa-5, respectively (Figure 3) [4]. In the phylogenetic tree, Shisa-1, Shisa-2, and Shisa-3 were in the same clade (Figure 1), while Shisa-4 and Shisa-5 were in the same clade (Figure 1). Therefore, “[C/Y]CC[KQT]C” might be the clade signature for shisa-1–3, while “*C**C[SP]CC” might be the clade signature for Shisa-4 and Shisa-5. *X. laevis* Shisa-2 and Shisa-3 are ER-specific proteins [6], while Shisa-1 is expressed as both an ER-residing and a secreted form [5]. No ER retention signal exists in these three *shisa* subfamily proteins [5,6], and their location in ER is the key to their mediation of cellular signaling [5,6,7]. Both the amino half of Shisa-1, which includes these cysteine residues and the conserved cysteine-rich domain, and the left carboxy half are important mediators of the cellular signaling in *X. laevis* [5]. Thus, the ER retention signals in these three *shisa* subfamily proteins might scatter at different locations not only in the amino half but also in the carboxy half. A comparative analysis of Shisa-1 and Shisa-2/3 protein sequences might give us a clue. However, the conserved cysteine-rich domain of mouse SHISA-5 was required for its interaction with other proteins, and the proline-rich region in the carboxy half was for its ER-specific location [16]. This suggested that both the amino and the carboxy halves were essential for its effective functioning in the correct position, which could also be why only half of *X. laevis* Shisa-1 did not correctly mediate its signal transmission function alone [5]. However, we cannot exclude the cysteine residues of the clade feature from the interaction between these *shisa* proteins and other proteins or the ER-specific expression because they were retained in the process of protein–protein interactions and ER-specific expression analysis. In zebrafish Shisa-1–3, a region with multiple prolines that was not accumulated similarly mouse SHISA-5 was observed. Further studies are needed to elucidate whether these proline regions play the same roles as mouse SHISA-5. An analysis of these conserved sequences might help to explain the functional conservation of these genes among different species as well as the similarities and differences between them and other subfamily genes.

Functional divergence after gene duplication for *shisa* family proteins in vertebrates has divergent C-terminal regions [4]. In zebrafish, PY motifs are present in their C-terminal regions of Shisa-3, 4, 5, and 7, and a PY-like motif, “PLSY” and “PTGY” in Shisa-6 and 8, respectively (Figure 3). As the PY motif can interact with NEDD4 family proteins, zebrafish Shisa-3, 4, 5, and 7 may be adaptor proteins that regulate the ubiquitination and degradation of other target proteins [4]. Future studies should analyze whether the function of the PY-like motif is the same as that of the PY motif. The PDZ-domain interacting motif ([ST] ×[VLI]) “VTV” or “VTI” is present at the C-terminus of zebrafish Shisa-1 and Shisa-7–9, which is a “VTM” in shisa-2. Many proteins containing PDZ-domain play a central role in scaffolding macromolecular complexes, which is critical to signaling and trafficking pathways [26,27]. As a result, the function of zebrafish Shisa-1 and Shisa-7–9 might be via their PDZ-domain interacting motif. Whether zebrafish Shisa-2 functions through the “VTM” needs to be studied further. In zebrafish Shisa-6, no PDZ-domain interacting motif is present, which is different from that in other species [4]. The function of zebrafish Shisa-6 may differ from that of other species, or the PDZ-domain interacting motif may not play a role in shisa-6’s functional implementation. In zebrafish Shisa-7, a GRID domain is present, which is critical for the interaction between SHISA-7 and the GABA_A_ receptor, further promoting GABA_A_ receptor trafficking to the cell surface in the mouse brain [12]. Zebrafish Shisa-7 might, therefore, be important for neurological development.

### 3.3. Extensive Participation of Shisa Family Genes during Zebrafish Embryonic Development

*Shisa* plays various roles in developmental processes [4]. The expression patterns of *shisa* family genes suggest they might play different roles during embryonic development. *Shisa-3* was expressed highly at 0.2 hpf, the first cell stage, and 16 hpf. At the first cell stage, cytoplasmic movements begin [28], and the first cell appears. At 16 hpf, the peripheral and central sensory axons extend to their destination location [28]; therefore, *shisa-3* might be critical to these developmental processes. Notably, *shisa-9b*, which might originate from fish-specific genome duplication, was expressed from 12 hpf. The Kupffer’s-vesicle appeared at approximately this time (11.7 hpf) [28]. Whether this gene represents the formation of Kupffer’s-vesicle still needs to be investigated. The semi-quantitative PCR results suggested that zebrafish *shisa* family genes might be involved in different development processes; however, the actual participation process should be determined through spatial expression and functional analysis in the future.

### 3.4. Shisa-2 Regulating the Convergent Extension Cell Movement of Somitic Precursors in Zebrafish

The results of in situ hybridization demonstrated that zebrafish *shisa-2* was expressed in the presomitic mesoderm (PSM), somites, optic vesicles, gill arches, and head regions. The expression is similar to that of *X. laevis*, chicken, and mouse *shisa-2* [6,15,17,29]. The conserved expression in somite and PSM suggested that *shisa-2* might be involved in the somite formation. Indeed, the convergent extension cell movement of somitic precursors and the maturation of somitic precursors are affected by *shisa-2* in *X. laevis* [6]. The effect of zebrafish *shisa-2* on the somite formation was preliminarily analyzed through knockdown. In wild-type zebrafish, *mesp-ab* is expressed as one or two stripes in the front compartment of potential somites in the anterior presomitic mesoderm [30]. In the *shisa-2* knockdown zebrafish, the space between the left and right stripes of the *mesp-ab* expression was wider than that in the control, and the expression was scattered and not clustered as a stripe. This result indicated that the convergent extension cell movement of somitic precursors was delayed after *shisa-2* expression was downregulated. Therefore, *shisa-2* may be crucial in the convergent extension cell movement of somitic precursors in zebrafish. The function of *shisa-2* might be conserved between zebrafish and African clawed frogs. In *X. laevis*, Shisa-2 regulated proper segmental patterning through individual inhibition of Wnt and FGF signaling [6], while in C2C12 myoblast, SHISA-2 promoted myoblast fusion via Rac1/Cdc42-mediated cytoskeletal F-actin remodeling [8]. The exact in vivo function and mechanism of *shisa-2* in zebrafish need to be explored in the future.

### 3.5. Limitations

The expression of the *shisa* family genes suggested that all of them might be crucial in embryonic development. We could not confirm whether they have a tissue- or cell-specific expression pattern and function. As a result, the spatiotemporal expression should be investigated using other methods, such as in situ hybridization, in the future. The function of *shisa-2* was only preliminarily analyzed through the recently established Cas13d-mediated gene knockdown in the embryonic stage. The method still has shortcomings, such as a lack of efficiency detection and specificity analysis methods. Thus, effective and specific detection methods should be discovered in the future. A homozygous mutant with a *shisa-2* knockout should also be used to analyze its function in the future.

## 4. Materials and Methods

### 4.1. Sample Collection

Wild-type zebrafish (TU strain) were cultured in a recirculation culture system at the institute aquarium (temperature: 28.5 ± 1 °C; light/dark cycle = 14 h/10 h). Fish were fed twice daily with commercial particulate food and once daily with brine shrimp. Fertilized eggs were obtained by mixing one male and two female fish in the morning. After washing with cycling water several times, fertilized eggs of the zebrafish were collected in a Petri dish ready for microinjection or sample collection.

For sample collection, the embryos were incubated in an incubator at 28.5 ± 1 °C, obtained at different developmental stages. Approximately 30 embryos were collected in a centrifugation tube for each sample, immediately frozen in liquid nitrogen, and stored at −80 °C until RNA extraction. The wild-type zebrafish embryos and the embryos injected with Cas13d mRNA and gRNA at 12 hpf were fixed in 4% paraformaldehyde (prepared in 1× phosphate-buffered saline (PBS)) overnight at 4 °C. The embryos were dehydrated and stored in 100% methanol for in situ hybridization.

### 4.2. Total RNA Isolation and cDNA Synthesis

Each sample was placed in a centrifugation tube containing 200 μL of TRIzol Reagent (Toroivd Tech. Comp., Shanghai, China) and homogenized using a grinding rod. Total RNA was extracted according to the manufacturer’s instructions. The quality of isolated RNA was checked using 1.5% agarose gel electrophoresis, and its concentration and purity were determined using a NanoDrop 2000 spectrophotometer (ThermoFisher Scientific, Waltham, MA, USA). For each sample, 1 μg of RNA was transcribed into cDNA using the TransScript^®^ One-step gDNA Removal and cDNA Synthesis SuperMix kit (TransGen Biotech, Beijing, China) with OligdT.

### 4.3. Cloning of Zebrafish Shisa Family Genes

All *shisa* family genes were downloaded from the annotated zebrafish genome in the National Center for Biotechnology Information (NCBI) database (https://www.ncbi.nlm.nih.gov/ (accessed on 15 February 2022)). Primers were designed based on the predicted ORF sequences (Table 3). PCR was performed using GoldStar Taq DNA Polymerase (CWBIO, Ltd., Beijing, China) and a mixed cDNA template of different developmental stages of zebrafish embryos. The PCR conditions were as follows: 5 min at 94 °C, 35 cycles of 5 s at 94 °C, 30 s at 50–60 °C, 2 min at 72 °C, and a final 5 min extension at 72 °C. All genes were cloned, ligated into TOPO vectors, and sequenced.

### 4.4. Bioinformatic Analysis

Phylogenetic analysis was performed using the amino acid sequences of the *shisa* family genes from *H. sapiens*, *M. musculus*, *G. gallus*, *X. laevis*, *X. tropicalis*, and *D. rerio* (Table 1). A Maximum likelihood tree was constructed using MEGA 7.0 with 1000 bootstrap replicates [18]. All amino acid sequences of zebrafish Shisa were aligned, and the average amino acid identity was calculated using DNAMAN 11.0 (http://www.lynnon.com (accessed on 14 February 2023)). For Synteny analysis, TBtools [31] was used to obtain the location of the target gene in *H. sapiens*, *M. musculus*, *G. gallus*, *X. tropicalis*, and *D. rerio*, and all of the gene information, such as gene type and location, were visualized using the ggplot2 tool [32] of R programming software (version 4.2.2). Based on the zebrafish genomic sequence annotation (GRCz11), the gene structure was revealed in TBtools [31]. The motif, including the signaling peptide, was predicted using the SMART online software (http://smart.embl-heidelberg.de/ (accessed on 19 October 2022)) combined with the Pfam, SignalP, and TMHMM databases. The predicted structural domains were visualized using the IBS software (Illustrator for Biological Sequences, version 1.0).

### 4.5. Knockdown of Shisa-2 in Zebrafish Embryos Using Cas13d mRNA and gRNAs

Zebrafish *shisa-*2 was knockdown using Cas13d mRNA and gRNAs as described by Kushawah et al. [33]. The Cas13d ORF fragment was cloned using primers (Table 4, Cas13D-F-psp64-T7/Cas13D-R-psp64-T7) with a T7 promoter in the 5′-end and the KOD enzyme. After purification, the PCR product was recombined into the *Sal* I and *BamH* I sites of the psp64 polyA vector using the EZ clone and named Cas13d/psp64. The Cas13d/psp64 was linearized using *Pvu* II (Takala, Kusatsu, Japan) and purified by phenol/chloroform as a template to synthesize the Cas13d mRNA using mMESSAGE mMACHINE™ T7 Transcription Kit (ThermoFisher Scientific, Waltham, MA, USA). Four guide RNAs (gRNA 1–4) were designed using online software (https://cas13design.nygenome.org/ (accessed on 6 September 2021)) [34,35]. The gRNA DNA template was generated by PCR using *pfu* enzyme and primers (Table 4; Cas13d-Universal-F and Cas13D-zfshisa2- gRNA1–4). After purification with phenol/chloroform, the PCR products were used as templates to synthesize gRNA using a TranscriptAid T7 High Yield Transcription Kit (ThermoFisher Scientific). Following synthesis, the Cas13d mRNA and gRNAs were purified using SigmaSpin™ Post-Reaction Clean-Up Columns (Sigma Aldrich, St. Louis, MO, USA).

At one cell stage, the optimized concentration of Cas13d mRNA (200 ng/μL) and gRNAs (total 800 ng/μL, 200 ng/μL for each) together, Cas13d mRNA (200 ng/μL), or gRNAs (total 800 ng/μL, 200 ng/μL for each) in 0.2M KCl were microinjected into the cell of zebrafish embryo using a microinjector (Pli-100; Harvard Apparatus, Holliston, MA, USA).

At 48 hpf, after the chorion was removed, the embryos were observed in water under a microscope (DM LB2, Wetzlar, Germany) and photographed with a WeiTu HTC2000 digital camera; the number was counted under a stereoscope (Leica 12.5, Wetzlar, Germany).

### 4.6. Rescue Using Flounder Shisa-2 mRNA

Flounder *shisa-2* was cloned using primers (Table 5, floundershisa2-F/R) and ligated into pEASY-T3, which was termed as flounder-shisa2/T3. After the sequence had been confirmed, the open read frame was cloned using primers (flounder-shisa2psp64-F/R) with the *pfu* enzyme and the flounder-shisa2/T3 plasmid as the template. After purification, the PCR product was recombined into the *Sal* I sites of the psp64 polyA vector using the EZ clone and named floundershisa2/psp64. The flounder-shisa2/psp64 was linearized using *Xba* I (Takala) and purified by phenol/chloroform as a template to synthesize the flounder *shisa-2* RNA using mMESSAGE mMACHINE™ Sp6 Transcription Kit (ThermoFisher Scientific).

At one cell stage, the optimized concentration of Cas13d mRNA (200 ng/μL) and gRNAs (total 800 ng/μL, 200 ng/μL for each) with or without flounder *shisa-2* mRNA (24 ng/μL) in 0.2 M KCl were microinjected into the cells of zebrafish embryos.

At 48 hpf, the embryos were treated, observed, photographed, and counted as knockdown (Section 4.5).

### 4.7. Semi-Quantitative PCR

The primers (Table 6) used for semi-quantitative PCR of zebrafish *shisa* genes were designed using Primer3 (https://www.primer3plus.com/index.html (accessed on 2 June 2022)) and Primer-BLAST of NCBI (https://www.ncbi.nlm.nih.gov/tools/primer-blast/ (accessed on 2 June 2022)). For the semi-quantitative PCR, *ef-1a* was used as an internal reference gene. The total PCR volume was 25 μL and included different amounts of cDNA templates, 12.5 μL of 2 × Taq Master Mix (Vazyme, China), 1 μL of forward primer, 1 μL of reverse primer, and 9.5 μL of ddH_2_O. The volume of cDNA templates in the other gene response systems was 2 μL, except for *shisa-3*, *shisa-4*, and *shisa-9b*, which had an amount of 4 μL because their expressions were very weak. The PCR protocol was as follows: 95 °C for 3 min, 94 °C for 15 s, 55 °C for 15 s, 72 °C for 15 s, 35 cycles, and finally, 72 °C for 5 min. All RT–PCR experiments were performed in triplicate. The corresponding plasmids obtained during gene isolation (Section 4.3) were used as a positive control.

### 4.8. Whole Mount In Situ Hybridization

Whole-mount in situ hybridization *was* performed by modifying the method described by Du and Dienhart [36]. Briefly, digoxigenin-labeled RNA probes were synthesized against *shisa-2* and *mesp-ab* mRNA. Subsequently, the fixed embryos were dechorionated, rehydrated in 50% methanol in PBST for 5 min, and washed with PBST (1× PBS pH 7.4 + 0.1% Tween 20) for 5 min. The embryos were soaked in the prehybridization buffer for 4 h at 65 °C for prehybridization and incubated in a hybridization buffer with each RNA probe (100–200 ng) overnight. A nitroblue tetrazolium chloride (NBT)/5-bromo-4-chloro-3-indolyl phosphate (BCIP) substrate was added to detect alkaline phosphatase until a color developed, and the reaction was stopped by rinsing with PBST. The embryos were photographed in glycerol under a microscope (Leica DM LB2) with a WeiTu HTC2000 digital camera.

## Figures and Tables

**Table 1:** *Shisa* genes used in Maximum likelihood tree construction.

Gene Name (NCBI Name)	Species	GenBank Accession Number
*Shisa-like 1b*	*Danio rerio*	XM_002667571.6
*Shisa-1* (*shisa 2a*)	*Danio rerio*	NM_001003631.1
*Shisa-2*	*Danio rerio*	XM_003201303.5
*Shisa-3*	*Danio rerio*	NM_001080662.2
*Shisa-4*	*Danio rerio*	NM_001017869.1
*Shisa-5*	*Danio rerio*	NM_001044870.1
*Shisa-6*	*Danio rerio*	XM_002667291.5
*Shisa-7* (*shisa-7-like*)	*Danio rerio*	XM_021472968.1
*Shisa-8*	*Danio rerio*	XM_021480293.1
*Shisa-9a*	*Danio rerio*	NM_001013509.1
*Shisa-9b*	*Danio rerio*	NM_001135975.2
*Shisa-like 1*	*Gallus gallus*	XM_015291144.4
*Shisa-2*	*Gallus gallus*	NM_204501.2
*Shisa-3*	*Gallus gallus*	XM_040700477.2
*Shisa-4*	*Gallus gallus*	XM_015298798.4
*Shisa-5*	*Gallus gallus*	NM_001030591.3
*Shisa-6*	*Gallus gallus*	XM_025141681.2
*Shisa-8*	*Gallus gallus*	XM_003640380.6
*Shisa-9*	*Gallus gallus*	XM_040647667.2
*Shisa-2*	*Homo sapiens*	NM_001007538.2
*Shisa-3*	*Homo sapiens*	NM_001080505.3
*Shisa-4*	*Homo sapiens*	NM_198149.3
*Shisa-5*	*Homo sapiens*	NM_001272065.3
*Shisa-6*	*Homo sapiens*	NM_001173461.2
*Shisa-7*	*Homo sapiens*	NM_001145176.2
*Shisa-8*	*Homo sapiens*	NM_001207020.3
*Shisa-9*	*Homo sapiens*	NM_001145204.3
*Shisa-like 1*	*Homo sapiens*	NM_001099294.2
*Shisa-like 2a*	*Homo sapiens*	NM_001042693.3
*Shisa-like 2b*	*Homo sapiens*	NM_001164442.2
*Shisa-2*	*Mus musculus*	NM_145463.5
*Shisa-3*	*Mus musculus*	NM_001033415.3
*Shisa-4*	*Mus musculus*	NM_175259.5
*Shisa-5*	*Mus musculus*	NM_001284332.1
*Shisa-6*	*Mus musculus*	NM_001034874.4
*Shisa-7*	*Mus musculus*	NM_001290291.1
*Shisa-8*	*Mus musculus*	NM_001207021.2
*Shisa-9*	*Mus musculus*	NM_001174086.1
*Shisa-like 1*	*Mus musculus*	NM_001163145.2
*Shisa-like 2a*	*Mus musculus*	NM_001099303.2
*Shisa-like 2b*	*Mus musculus*	NM_029984.1
*Shisa-1*	*Xenopus tropicalis*	XM_004915754.4
*Shisa-2*	*Xenopus tropicalis*	XM_002940554.3
*Shisa-3*	*Xenopus tropicalis*	XM_002933451.5
*Shisa-4*	*Xenopus tropicalis*	XM_018091360.2
*Shisa-5*	*Xenopus laevis*	XM_018261282.2
*Shisa-6*	*Xenopus tropicalis*	XM_031894479.1
*Shisa-7*	*Xenopus tropicalis*	XM_002939964.4
*Shisa-8*	*Xenopus tropicalis*	XM_002934724.5
*Shisa-9*	*Xenopus tropicalis*	NM_001112925.1

**Table 2:** Percentage of phenotype at 48 hpf after *shisa-2* knockdown.

Group	Phenotype (%) (n/N)		
	First	Second	Third
Wild type	0 (0/59)	0 (0/80)	0 (0/102)
Cas13d mRNA Control	0 (0/103)	8.0 (2/25)	0 (0/48)
gRNAs Control	0 (0/83)	4.4 (2/45)	2.5 (1/40)
Cas13d mRNA + gRNAs	72.9 (70/96)	69.4 (34/49)	90.5 (19/21)

**Table 3:** Primers used in *shisa* gene cloning.

Gene Name (NCBI Name)	Primer Name	Sequence (5′-3′)	GenBank Number
*shisa-like 1*	shisa like-1-F	CTGATGGAGGACAAGAAGATG	XM_002667571.6
	shisa like-1-R	CTATGGTCAGTCTCAGGCT	
*shisa-1* (*Shisa 2a*)	shisa-2a-F	AAGATGAAGTCATCGGCATC	NM_001003631.1
	shisa-2a-R	AATAATCCATGTGTAGTCC	
*shisa-2*	shisa-2-F	GTGGTTTGTGACACGATG	XM_003201303.5
	shisa-2-R	CATTGGGTTTCACATGGT	
*shisa-3*	shisa-3-F	AATTCAAGTTTGTCGGCGAG	NM_001080662.2
	shisa-3-R	GAGGTCACAGGTCAGCTCTG	
*shisa-4*	shisa-4-F	GATGTCCTTCTACGCTGTC	NM_001017869.1
	shisa-4-R	GTTATCTTCTCCTCGCAGAG	
*shisa-5*	shisa-5-F	GCGAGAGAGCAGCGCTATG	NM_001044870.1
	shisa-5-R	AAATGAACCATCCAGCTTGT	
*shisa-6*	shisa-6-F	GAAACACACCCTGAAGCCAT	XM_002667291.5
	shisa-6-R	TCCAGAGCATCCAAACAGC	
*shisa-7* (*shisa-7-like*)	shisa-like-7-F	CATGTAAAGATGATGCCCACC	XM_021472968.1
	shisa-like-7-R	CCTCTACCATCCTCCAACTC	
*shisa-8b*	shisa-8b-F	ATTTCTGGACAGGACCAGAG	XM_021480293.1
	shisa-8b-R	TGCATACAGTTATCTGAGTC	
*shisa-9a*	shisa-9a-F	CCAGGAGACTACAGGATGA	NM_001013509.1
	shisa-9a-R	TCCCGCTCTCAGCTGCTTC	
*shisa-9b*	shisa-9b-F	CCTCAAACATGAGCAGCATC	NM_001135975.2
	shisa-9b-R	CCACGTTCACACAGTCACC	

**Table 4:** Primers used for the construction of Cas13d and synthesis of gRNA.

Primer Name	Sequence (5′-3′)	Purpose
Cas13D-F-psp64-T7	AAGCTTGGGCTGCAGGTCGACTAATACGACTCACTATAGGGAGCCACCATGAGCGAGGCCAGCATCGAAAAAAAAAAG	construction of Cas13d
Cas13D-R-psp64-T7	TGGGAGCTCGCCCGGGGATCCTTAAGCGTAATCTGGAACATCGTATGGGTAAGCGGCCGCTCCGGATCCGGAATTGCCG	construction of Cas13d
Cas13d-Universal-F	TAATACGACTCACTATAGGAACCCCTACCAACTGGTCGGGGTTTGAAAC	synthesis of gRNA
Cas13D-zfshisa2-gRNA1	ATCGTCGGCTCAGTTTTTGTGGCGTTTCAAACCCCGACCAGTTGGTAGGGGTT	synthesis of gRNA
Cas13D-zfshisa2-gRNA2	TCGTCGGCTCAGTTTTTGTGGCAGTTTCAAACCCCGACCAGTTGGTAGGGGTT	synthesis of gRNA
Cas13D-zfshisa2-gRNA3	CGTCGGTTCAGTTTTTGTGGCATGTTTCAAACCCCGACCAGTTGGTAGGGGTT	synthesis of gRNA
Cas13D-zfshisa2-gRNA4	TGGGCTCTGTTGTTGCTGTATGCGTTTCAAACCCCGACCAGTTGGTAGGGGTT	synthesis of gRNA

**Table 5:** Primers used for flounder *shisa-2* cloning and mRNA synthesis.

Primer	Sequence (5′-3′)	Purpose
flounder-shisa-2-F	TGGTCGAGGATGTGGGGCGG	flounder *shisa-2* cloning
flounder-shisa-2-R	GTGGCAGAGTGGACTACATG	flounder *shisa-2* cloning
flounder-shisa2psp64-F	AAGCTTGGGCTGCAGGTCGACATGTGGGGCGGAGGTTTCCC	construction of flounder *shisa-2* mRNA expression vector
flounder-shisa2psp64-R	TGGGAGCTCGCCCGGGGATCCGTGGCAGAGTGGACTACATG	construction of flounder *shisa-2* mRNA expression vector

**Table 6:** Primers used for semi-quantitative PCR.

Gene Name	Primer Name	Sequence (5′-3′)
*shisa-like 1*	shisa-like-1-RT-F	ACTCTCGGACAACAAGACGT
	shisa-like-1-RT-R	CTATGGTCAGTCTCAGGCT
*shisa-1*	shisa-1-RT-F	CGGTGCGATTGTATCTGTCTG
	shisa-1-RT-R	AATAATCCATGTGTAGTCC
*shisa-2*	shisa-2-RT-F	AGTACCCATCTACGTGCCCT
	shisa-2-RT-R	GAGACTGTAACGGCCGGTAG
*shisa-3*	shisa-3-RT-F	CTGGACAGCAGTGGGAATTAC
	shisa-3-RT-R	TGTGAACATTGACCCATCGT
*shisa-4*	shisa-4-RT-F	GATGTCCTTCTACGCTGTC
	shisa-4-RT-R	TCATCGGATACTGAGGCACC
*shisa-5*	shisa-5-RT-F	GCGAGAGAGCAGCGCTATG
	shisa-5-RT-R	TGGGCTGATATGGTGGGTAC
*shisa-6*	shisa-6-RT-F	GAAACACACCCTGAAGCCAT
	shisa-6-RT-R	AGAGCAGGGTCATACGTGTC
*shisa-7*	shisa-7-RT-F	CATGTAAAGATGATGCCCACC
	shisa-7-RT-R	CAGGTCCCACAGCAGTAGAT
*shisa-8*	shisa-8-RT-F	TGCAAACCGGAGCTACTACA
	shisa-8-RT-R	TGCATACAGTTATCTGAGTC
*shisa-9a*	shisa-9a-RT-F	CCAGGAGACTACAGGATGA
	shisa-9a-RT-R	TATCCCAACCAGTGCCATGA
*shisa-9b*	shisa-9b-RT-F	TCACCCCTATGAGCCGTC

## Data Availability

All data generated or analyzed during this study are included in this published article and the Supplementary File.

## Supplementary Materials

The supporting information can be downloaded at: https://www.mdpi.com/article/10.3390/ijms241814062/s1.
