# Supplementary material for: Characteristics of Shisa Family Genes in Zebrafish
Source: Int J Mol Sci. 2023 Sep 14;24(18):14062. doi: 10.3390/ijms241814062 (PMC10531659; doi:10.3390/ijms241814062)
Supplement: Supplementary file 1 [file ijms-24-14062-s001.zip › ijms-2509123-supplementary.pdf]

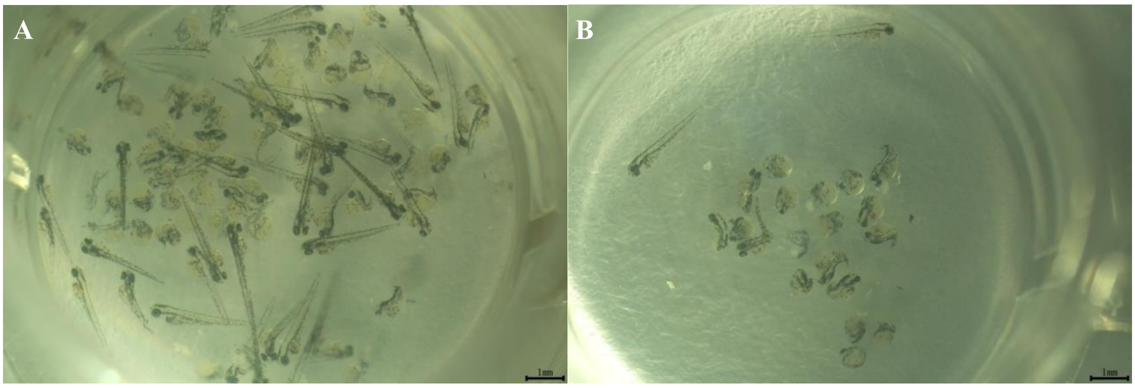

Figure S1. Rescue experiment 1.

A, Rescue Phenotype of zebrafish embryos (48 hpf) by co-injection with Cas13d mRNA, gRNAs and flounder shisa-2 mRNA (Abnormal (weak): Normal = 65(8):33); B, Control, Knockdown Phenotype of zebrafish embryos (48 hpf) after shisa-2 knockdown by co-injection with Cas13d mRNA and gRNAs (Abnormal (weak): Normal: = 19(1): 2).

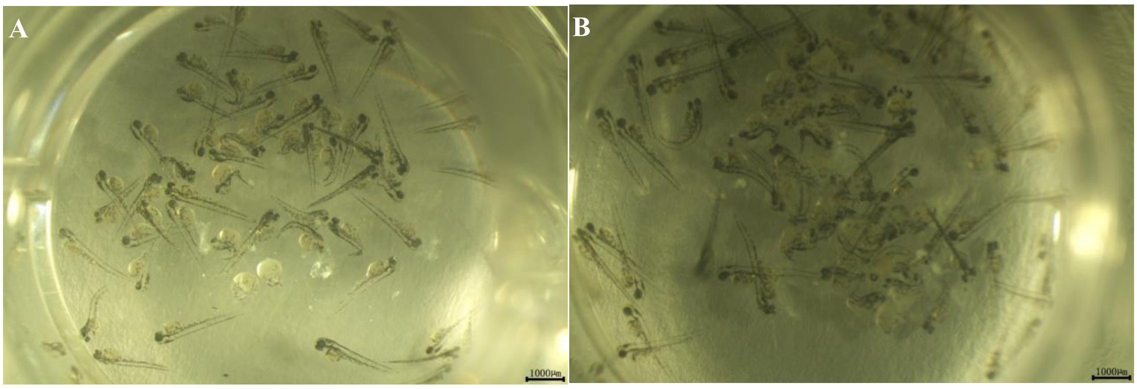

Figure S2. Rescue experiment 2.

A, Rescue Phenotype of zebrafish embryos (48 hpf) by co-injection with Cas13d mRNA, gRNAs and flounder shisa-2 mRNA, (Abnormal (weak): Normal: =15(6): 45); B, Control, Phenotype of zebrafish embryos (48 hpf) after shisa-2 knockdown by co-injection with Cas13d mRNA and gRNAs, (Abnormal (weak): Normal: = 39(4): 48).
